# Radiographic and visual response to the type II RAF inhibitor tovorafenib in children with relapsed/refractory optic pathway glioma in the FIREFLY-1 trial

**DOI:** 10.1093/neuonc/noae274

**Published:** 2024-12-19

**Authors:** Karsten Nysom, Lindsay B Kilburn, Sarah E S Leary, Daniel B Landi, Evelien de Vos-Kerkhof, Sébastien Perreault, Olaf Witt, David S Ziegler, Pablo Hernáiz Driever, Andrea T Franson, Patricia A Baxter, Nicholas S Whipple, Cassie Kline, Devorah Segal, Nada Jabado, Simon Bailey, Geoffrey McCowage, Jordan R Hansford, Dong-Anh Khuong-Quang, Nicholas G Gottardo, Timothy Hassall, Jung Woo Han, Michal Yalon Oren, Susan N Chi, Jiaheng Qiu, Daniel Da Costa, Sandya Govinda Raju, Peter Manley, Darren Hargrave

**Affiliations:** Department of Pediatrics and Adolescent Medicine, Copenhagen University Hospital, Rigshospitalet, Copenhagen, Capital Region of Denmark, Denmark; Children’s National Hospital, Washington, District of Columbia, USA; Cancer and Blood Disorders Center, Seattle Children’s Hospital, Seattle, Washington, USA; Department of Pediatrics, Duke University, Durham, North Carolina, USA; Princess Máxima Center for Pediatric Oncology, Utrecht, The Netherlands; CHU Sainte-Justine, Université de Montréal, Montreal, Quebec, Canada; National Center for Tumor Diseases (NCT), Heidelberg, Germany; German Cancer Consortium (DKTK), Heidelberg, Germany; Department of Pediatric Oncology, Hematology, Immunology and Pulmonology, Heidelberg University Hospital, Heidelberg, Germany; Clinical Cooperation Unit Pediatric Oncology, German Cancer Research Center (DKFZ), Heidelberg, Germany; Hopp Children’s Cancer Center Heidelberg (KiTZ), Heidelberg, Germany; School of Clinical Medicine, University of New South Wales, Sydney, New South Wales, Australia; Children’s Cancer Institute, Lowy Cancer Research Centre, University of New South Wales, Sydney, New South Wales, Australia; Kids Cancer Centre, Sydney Children’s Hospital, Randwick, New South Wales, Australia; Department of Pediatric Oncology/Hematology, Charité-Universitätsmedizin Berlin, Corporate Member of Freie Universität Berlin and Humboldt-Universität Berlin, German HIT-LOGGIC-Registry for LGG in Children and Adolescents, Pediatric Neurooncology Program, Berlin, Germany; Department of Pediatrics, C.S. Mott Children’s Hospital, University of Michigan Medical School, Ann Arbor, Michigan, USA; Texas Children’s Cancer Center, Texas Children’s Hospital, Baylor College of Medicine, Houston, Texas, USA; Primary Children’s Hospital and University of Utah, Salt Lake City, Utah, USA; Division of Oncology, Department of Pediatrics, Children’s Hospital of Philadelphia, University of Pennsylvania Perelman School of Medicine, Philadelphia, Pennsylvania, USA; NYU Langone Health, New York, New York, USA; Department of Pediatrics, McGill University Health Centre (MUHC), The Montreal Children’s Hospital (MCH), Montréal, Quebec, Canada; Great North Children’s Hospital and Newcastle University Centre for Cancer, Newcastle-upon-Tyne, UK; Sydney Children’s Hospitals Network, Westmead, New South Wales, Australia; South Australian Immunogenomics Cancer Institute, University of Adelaide, Adelaide, Australia; South Australia Health and Medical Research Institute, Adelaide, South Australia, Australia; Michael Rice Centre for Hematology and Oncology, Women’s and Children’s Hospital, Adelaide, South Australia, Australia; Children’s Cancer Centre, The Royal Children’s Hospital Melbourne, Melbourne, Victoria, Australia; Brain Tumor Research Program, Telethon Kids Cancer Centre, Telethon Kids Institute, University of Western Australia, Perth, Washington, Australia; Department of Pediatric and Adolescent Oncology and Hematology, Perth Children’s Hospital, Perth, Western Australia, Australia; Children’s Health Queensland Hospital and Health Service, South Brisbane, Australia; Severance Hospital, Yonsei University Health System, Seoul, Republic of Korea; Pediatric Hemato-Oncology Department, Sheba Medical Center, Ramat Gan, Tel Aviv District, Israel; Pediatric Neuro-Oncology, Dana-Farber/Boston Children’s Cancer and Blood Disorders Center, Boston, Massachusetts, USA; Day One Biopharmaceuticals, Brisbane, California, USA; Day One Biopharmaceuticals, Brisbane, California, USA; Day One Biopharmaceuticals, Brisbane, California, USA; Day One Biopharmaceuticals, Brisbane, California, USA; UCL Great Ormond Street Institute of Child Health and Great Ormond Street Hospital for Children, London, UK

**Keywords:** BRAF, FIREFLY-1, optic pathway glioma, tovorafenib, visual acuity

## Abstract

**Background:**

Due to their anatomical locations, optic pathway gliomas (OPGs) can rarely be cured by resection. Given the importance of preserving visual function, we analyzed radiological and visual acuity (VA) outcomes for the type II RAF inhibitor tovorafenib in the OPG subgroup of the phase 2 FIREFLY-1 trial.

**Methods:**

FIREFLY-1 investigated the efficacy (arm 1, *n* = 77), safety, and tolerability (arms 1/2) of tovorafenib (420 mg/m^2^ once weekly; 600 mg maximum) in patients with *BRAF*-altered relapsed/refractory pediatric low-grade glioma (pLGG). In this post hoc analysis, anti-tumor activity and VA were analyzed in arm 1 patients with OPG. Anti-tumor activity was independently assessed per Response Assessment in Neuro-Oncology high-grade glioma (RANO-HGG), Response Assessment in Pediatric Neuro-Oncology-LGG (RAPNO), and RANO-LGG criteria. The data cutoff was June 5, 2023.

**Results:**

Forty-two of 77 patients had OPGs; 35 of 42 had ≥2 VA assessments. The overall response rate in the OPG subgroup according to RANO-HGG, RAPNO, and RANO-LGG criteria were 64%, 50%, and 55%, with clinical benefit rates of 95%, 88%, and 90%, respectively. VA per patient was preserved for 80% of patients; 31% demonstrated improved VA; VA per eye was preserved in 87%, with 27% improving. The safety profile in the arm 1 OPG subgroup was similar to the overall FIREFLY-1 safety analysis set.

**Conclusions:**

Tovorafenib demonstrated anti-tumor activity in relapsed/refractory *BRAF*-altered OPG across radiological assessment criteria and was generally well tolerated. Importantly, vision remained stable or improved in most patients.

**Key Points**

- Tovorafenib may be an effective therapy for *BRAF*-altered optic pathway glioma.
- Stable or improved vision per patient and per eye was seen in 80% of patients and 87% of eyes, respectively.
- Preservation of vision is an important treatment outcome in optic pathway glioma.

**Importance of the Study**

Subgroup analysis of the FIREFLY-1 trial showed that the type II RAF inhibitor tovorafenib achieved clinically meaningful, durable, rapid tumor responses in patients with *BRAF*-altered relapsed/refractory optic pathway glioma (OPG), according to both contrast-enhancement-based and T2/fluid-attenuated inversion recovery-based assessment criteria. Importantly, visual acuity (VA) remained stable or improved for most patients: VA per patient and per eye improved in 31% and 27% of patients/eyes, and remained stable in 49% and 60%, that is, visual preservation in 80% of patients and 87% of eyes. VA changed regardless of baseline VA (blind eyes excluded) and time from primary diagnosis. Furthermore, improvements in VA occurred in some patients with only small maximal changes in tumor size. These data suggest that tovorafenib may offer an important new treatment option for patients with *BRAF*-altered, relapsed/refractory OPG. The phase 3 LOGGIC/FIREFLY-2 (NCT05566795) trial of tovorafenib versus standard-of-care chemotherapy in patients with pediatric low-grade glioma requiring front-line systemic treatment is ongoing.

Low-grade glioma (LGG) is the most common central nervous system (CNS) tumor in children.^1^ Approximately one-third of pediatric (p)LGGs are located in the optic pathway/hypothalamic region and are referred to as optic pathway gliomas (OPGs).^2,3^ While pLGGs located in the posterior fossa or the cerebral hemispheres are potentially curable by surgical resection, any resection of OPGs will often cause functional deterioration, including worsened visual, endocrinologic, and/or motor deficits.^2,4^ Patients with OPG requiring therapy, usually due to a growing tumor and/or threatened vision, are most frequently treated with systemic therapy. Such children often require several lines of therapy over the first 2 decades of life, which can lead to significant morbidities, while tumor progression can potentially cause or worsen functional deficits.^4,5^

Up to 50% of OPGs arise in patients with the autosomal dominant tumor predisposition syndrome, neurofibromatosis type 1 (NF1), with symptomatic tumors typically occurring before 6 years of age.^3,6,7^ OPGs that are sporadic in nature (ie, not associated with NF1), are more likely to progress, and more frequently require therapeutic intervention.^2,8^ While a subset of children with OPG may not require active intervention, anticancer treatment is generally warranted for patients with visual deterioration in order to stabilize or prevent further deterioration as visual function is key for their quality of survival and participation in age-appropriate activities.^8^ First-line treatment for children with OPG is generally comprised of chemotherapy, with radiotherapy avoided where possible due to a range of possible late effects including visual disturbance, endocrine deficiency, neurocognitive impairment, and secondary malignancy.^3,9,10^ The impact of chemotherapy on visual function in sporadic OPG is still unclear given the considerable heterogeneity of trial designs, treatment regimens, and outcome measures.^11^ However, the addition of bevacizumab to later lines of chemotherapy appears to be a promising approach, which may provide short-term disease control and result in visual improvement or preservation.^12,13^

As is the case for pLGGs in general, sporadic OPGs are commonly driven by oncogenic genomic alterations affecting *BRAF*, including *KIAA1549*::*BRAF* fusions and BRAF V600E point mutations.^14–17^ The ongoing phase 2 FIREFLY-1 (PNOC026; NCT04775485) trial is assessing the efficacy and safety of the investigational, oral, selective, CNS-penetrant type II RAF inhibitor, tovorafenib, in patients with *RAF*-altered relapsed/refractory pLGG or advanced solid tumors.^18^ Tovorafenib monotherapy achieved clinically meaningful, rapid, and durable tumor responses in children and young adults with *BRAF*-altered pLGG, as assessed by an independent radiology review committee (IRC) according to Response Assessment in Neuro-Oncology high-grade glioma (RANO-HGG),^19^ Response Assessment in Pediatric Neuro-Oncology-LGG (RAPNO),^20^ and RANO-LGG^21,22^ criteria. OPGs are the largest tumor location subgroup in the registrational arm of the FIREFLY-1 trial, representing a clinically important population of patients with sporadic relapsed/refractory pLGG.

While anti-tumor activity has traditionally been assessed by radiological response, there is an increasing appreciation of the importance of visual outcomes in patients with OPG and in using such functional endpoints in clinical trials, in some cases as the primary outcome.^23^ Consequently, we analyzed the efficacy of tovorafenib in the OPG subgroup of FIREFLY-1 arm 1, focusing on both radiological and visual acuity (VA) outcomes. We also describe the incidence of adverse events (AEs) in this subgroup and ophthalmologic AEs of special interest (AESI) in the trial safety analysis set of 137 patients with pLGG and the arm 1 OPG subgroup.

## Methods

### Trial Design

The design of the ongoing, open-label, 3-arm, phase 2 FIREFLY-1 (PNOC026; NCT04775485) trial of tovorafenib monotherapy in children, adolescents, and young adults with *RAF*-altered pLGGs or advanced solid tumors who have received at least 1 prior systemic therapy, has recently been described.^18^ Briefly, arm 1 of the trial enrolled patients with relapsed or refractory pLGG harboring an activating *BRAF* alteration, including BRAF V600 mutations and *KIAA1549*::*BRAF* fusions. Arm 2 of the trial is a pLGG expansion cohort, which provided treatment access for patients with *RAF*-altered pLGG following arm 1 closure. Arm 1 and Arm 2 are fully accrued. In the primary registrational analysis, efficacy was assessed in the 77 patients enrolled in arm 1, with safety assessed in all 137 treated patients in arms 1 and 2.

Tovorafenib was administered at the recommended phase 2 dose of 420 mg/m^2^ (not to exceed 600 mg) by mouth (tablet or liquid formulation), once weekly, in 28-day cycles.^18^ Treatment was continued until radiographic evidence of disease progression as determined by the treating investigator according to RANO-HGG criteria,^19^ unacceptable toxicity, decision to enter a drug holiday period, patient withdrawal of consent, or death. Patients with disease progression were allowed to continue tovorafenib treatment if they were deemed to be deriving clinical benefit from continuing trial treatment. Patients were treated for a planned period of 26 cycles, after which they could continue on tovorafenib or, at any point, opt to enter a drug holiday period. During this drug holiday period, patients could be retreated with tovorafenib if there was radiographic disease progression.

The trial was approved by an institutional review board/independent ethics committee at each trial site. The trial was conducted in accordance with current ethical principles and trial standards.^18^ All patients and/or their legally authorized representative provided written informed consent and pediatric assent before enrollment in the trial, according to local regulations.

### Eligibility

Full inclusion and exclusion criteria were recently published.^18^ Briefly, eligible patients in arm 1 were aged 6 months to 25 years, inclusive, with a histopathologically verified pLGG, which had previously been treated with at least 1 line of systemic therapy with subsequent evidence of radiographic progression, a documented known activating *BRAF* alteration and measurable disease as defined by RANO-HGG criteria, a Lansky (aged <16 years) or Karnofsky (aged ≥16 years) performance score of ≥50 and adequate organ function. Patients were excluded if their tumor harbored an additional known or expected to be activating molecular alteration; if they had symptoms of clinical progression without radiographically recurrent or radiographically progressive disease; a history or current evidence of central serous retinopathy, retinal vein occlusion, or ophthalmopathy present at baseline that would be considered a risk factor for either; clinically significant active cardiovascular disease; or if they were neurologically unstable despite adequate treatment. A known or suspected diagnosis of NF1 was an exclusion criterion.

### Trial Endpoints

The assessment of response in the primary analysis and the current subgroup analysis was undertaken using 3 different radiological response assessment criteria: RANO-HGG,^19^ which assesses tumor response primarily based on T1-weighted, contrast-enhanced imaging, and RAPNO^20^ and RANO-LGG,^21,22^ both of which assess tumor response primarily based on non-enhancing disease by T2/fluid-attenuated inversion recovery (FLAIR) sequences and include a minor response (MR) category. Patients were enrolled based on investigator-assessed measurable disease per RANO-HGG. Investigator response assessments per RANO-HGG were also the criteria on which cessation of treatment due to progressive disease was based. The response was subsequently analyzed according to all 3 radiological assessment criteria by blinded independent central review.

The primary endpoint in arm 1 was the overall response rate (ORR), calculated as the percentage of evaluable patients with the best overall confirmed response of complete response (CR) or partial response (PR), as assessed according to RANO-HGG criteria by an independent radiology review committee (IRC). Secondary endpoints included the ORR, as assessed according to RAPNO criteria by the IRC, and clinical benefit rate (CBR), progression-free survival, duration of response (DOR), and time to response (TTR), as assessed by the IRC using both RANO-HGG and RAPNO criteria. Post hoc exploratory endpoints for arm 1, added to the statistical analysis plan prior to the primary analysis at regulatory authority request, included ORR and CBR according to RANO-LGG criteria by IRC assessment. Per the FIREFLY-1 statistical analysis plan, ORRs for RAPNO and RANO-LGG calculations were defined as the percentage of evaluable patients with the best overall confirmed response of CR, PR or MR, and CBRs were calculated as the percentage of evaluable patients with the best overall confirmed response of CR, PR, MR, or stable disease (SD; calculated based on SD of any length of time and SD ≥12 months). The evaluation of changes in quality of life and health utilities measures was also an exploratory objective of the trial (to be reported at a later date).

### Assessments

Disease was assessed in arm 1 by MRI of the brain and spine at screening (up to 28 days prior to the first dose) and at the end of every 3 cycles thereafter. Safety assessments have been described previously.^18^ AEs considered of special interest (AESI) included rhabdomyolysis/myopathy, ventricular arrhythmias, intratumoral hemorrhage, secondary primary malignancies, ophthalmologic events, and decreased growth velocity. They were further reviewed and adjudicated for clinical relevance by the sponsor’s senior safety physician, and assessed for clinical relevance to the intended events of interest. Positively adjudicated ophthalmologic AESI are presented in this report. Patients with OPG or underlying visual function deficit related to their tumor had ophthalmology examinations performed at baseline, at the time of each radiographic disease assessment, and at the end of treatment. These examinations were performed by an ophthalmologist or other qualified site clinical personnel and included: a slit-lamp examination, specifically looking for corneal/lens abnormalities; a fundus examination with a comment on retinal abnormalities; visual fields to confrontation; optic disc appearance; and best-corrected VA (BCVA) as assessed per local institutional practice. Analyses of visual fields and optic nerve papillae were not routinely undertaken.

Functional VA assessments were age-specific and based on local standard practice; they included the use of Teller Acuity Cards^®^ (all patients), HOTV, or other Early Treatment Diabetic Retinopathy Study charts (in patients developmentally able to perform them)^24^; however, other methods were permitted. Assessments were done for each eye separately, at a recommended testing distance of 3 meters (or according to local standard practice). If the BCVA data derived at a particular visit were felt to be unreliable due to poor cooperation, testing was to be repeated 1-2 weeks later, and only the visit was believed to have yielded the most reliable data reported. The protocol recommended that to reduce variability in scoring, the same BCVA testing methodology should be used throughout the treatment period. BCVA was reported as a logarithm of the minimum angle of resolution (logMAR) score, where 0 is normal vision and positive values indicate reduced VA.

LogMAR range group categories used for the assessment of baseline VA status were adapted from Schulze-Bonsel et al,^25^ and Gnekow et al,^4^ and adjusted for age. *VA response* was assessed per eye and per patient, in accordance with the REiNS recommendations.^26^*VA response* per patient was defined according to outcome in both eyes from baseline to last follow-up; that is, for patients with VA assessments in both eyes: if VA improved in 1 eye and improved or was stable in the other eye, VA was classified as improved. Conversely, if VA improved or was stable in 1 eye but worsened in the other, VA was classified as having worsened. If VA was stable in both eyes VA was classified as stable. For patients with VA assessments in 1 eye only (blind in the other eye, with blind being defined as “no light perception” [logMAR ≥ 3.0]), the VA response was that of the single eye. A *confirmed VA response* was defined as a decrease from baseline by at least 0.2 logMAR at 2 consecutive visits, that is, every 3 cycles/~12 weeks apart. *Confirmed VA progressive disease* was defined as an increase from baseline of at least 0.2 logMAR at 2 consecutive assessments. VA was deemed to be stable if the criteria for VA response or VA progressive disease were not met. To evaluate the degree of clinical-radiological correlation across the different response assessment criteria, concordance between VA per patient and per eye and radiological outcomes from the start of tovorafenib treatment to the last follow-up were analyzed. Full concordance represented concordant outcomes for VA and radiological assessments; partial concordance represented either improvement (positive) or worsening (negative) of 1 parameter with the stability of the other; full discordance represented conflicting outcomes.

### Statistical Considerations

In this post hoc subgroup analysis, efficacy was analyzed in all patients in arm 1 with tumors classified as having optic pathway involvement. The evaluable populations for efficacy were as previously described for the primary analysis.^18^ Waterfall plots were generated for each patient’s best change in the sum of perpendicular diameters of measurable lesions. DOR was estimated using the Kaplan-Meier method. Patients with responses who had not progressed at data cutoff were censored at the date of their last adequate radiologic disease assessment. Analyses of outcomes in subgroups defined by baseline characteristics were not powered to enable statistical comparisons and were therefore purely descriptive. Safety assessments were based on the safety population in arms 1 and 2 and the OPG subgroup of arm 1. Statistical analyses were carried out using SAS v9.4. Analyses were based on a June 5, 2023 data cutoff.

## Results

### Patients and Disposition

Between May 6, 2021, and April 11, 2022, 42 patients with optic pathway tumor involvement were enrolled in arm 1. Their demographics and baseline characteristics are summarized in Table 1. The median age of patients was 8 years (range 2-16); most patients were male and white (57% each). A *KIAA1549*::*BRAF* fusion was identified in 81% of tumors, 7% had a chromosomal rearrangement involving *BRAF* (as detected by fluorescence in situ hybridization and presumed to represent a *KIAA1549*::*BRAF* or other *BRAF* fusion), and 12% had a BRAF V600E mutation. Patients had received a median of 3 lines of prior therapy (range 1-9); 69% had received a prior MEK and/or BRAF inhibitor. VA data are not included in the current analysis for 7 of the 42 patients: 4 had no VA assessments done due to bilateral blindness, 1 had no baseline visual assessment, 1 patient had no assessment after baseline, and 1 patient was deemed VA not evaluable despite scores being entered as the patient was uncooperative at each assessment. Of the remaining 35 patients, including 18 patients blind in 1 eye, baseline VA per eye (*n* = 52) was normal (up to 0.19) in 15 (29%), mildly impaired (0.2-0.5) in 20 (38%), moderately impaired (0.6-0.9) or severely impaired (1.0-1.3) in 6 (12%) each, profoundly impaired (1.4-1.6) in 1 (2%), counting fingers (1.7-2.0) in 3 (6%), and hand motion (2.1-2.4) in 1 (2%) (logMAR ranges^4,25^). The VA testing method used was consistent across all on-study assessments for 19 (54%) of 35 patients and varied between assessments in 16 (46%) of 35 patients (Supplementary Table S1).

**Table 1.** Patient and Baseline Characteristics

Characteristic	Arm 1 OPG Subgroup *n* = 42
Age, y	
Median (range)	8 (2-16)
Gender, *n* (%)	
Male	24 (57)
Female	18 (43)
Race, *n* (%)^a^^,^^b^	
White	24 (57)
Asian	2 (5)
Black	1 (2)
Multiple	2 (5)
Other	3 (7)
Not specified	10 (24)
Ethnicity, *n* (%)	
Hispanic or Latino	2 (5)
Not Hispanic or Latino	29 (69)
Not stated	10 (24)
Missing	1 (2)
BRAF alteration, *n* (%)	
* BRAF* fusion^c^	37 (88)
* KIAA1549*::*BRAF* fusion	34 (81)
* *Other	3 (7)
BRAF V600E mutation	5 (12)
Baseline Lansky performance score, *n/n* (%)^d^	
50-70	1/41 (2)
80-100	40/41 (98)
Number of prior lines of systemic therapy, *n* (%)	
Median (range)	3 (1-9)
1	5 (12)
2	11 (26)
≥3	26 (62)
Prior MAPK pathway targeted therapy, *n* (%)^e^	
Prior MEK inhibitor	28 (67)
Prior BRAF inhibitor	3 (7)
Prior MEK and BRAF inhibitors	2 (5)
Prior MEK and/or BRAF inhibitor	29 (69)
Any prior surgery for primary disease, *n* (%)	
Pre-operative staging	
Localized disease	35 (83)
Disseminated/metastatic disease	4 (10)
Leptomeningeal spread	3 (7)
Post-operative staging^f^	
Subtotal resection	13 (31)
Biopsy only, resection not attempted	29 (69)
Prior radiotherapy *n* (%)	1 (2)
Patients with bilateral blindness at enrollment (vision not tested), *n*	4
Visual acuity at enrollment, (logMAR range),^g^*n* (%)	Per eye *n* = 52
Normal (up to 0.19)	15 (29)^g^
Mild impairment (0.2-0.5)	20 (38)
Moderate impairment (0.6-0.9)	6 (12)
Severe impairment (1.0-1.3)	6 (12)
Profound/worse impairment (1.4-1.6)	1 (2)
Counting fingers (1.7-2.0)	3 (6)
Hand motion (2.1-2.4)	1 (2)
Light perception (2.5-2.9)	0
No light perception (≥3.0)	0

Abbreviations: OPG, optic pathway glioma; logMAR, logarithm of the minimum angle of resolution; MAPK, mitogen-activated protein kinase.

^a^There were no Native Hawaiian or other Pacific Islander, American Indian, or Alaska Native participants.

^b^None were missing.

^c^Includes 3 patients with *BRAF* rearrangement per fluorescence in situ hybridization.

^d^Denominator for Lansky performance score is the number of patients <16 years of age. There was only 1 patient ≥16 years of age; their baseline Karnofsky performance score was assessed as 80-100. The baseline is defined as the last available assessment prior to the start of tovorafenib on cycle 1 day 1.

^e^Patients who had previously received both a MEK inhibitor and also a BRAF inhibitor are recorded in both the “Prior MEK inhibitor” and “Prior BRAF inhibitor” groups.

^f^No gross total resections.

^g^Includes a 2-year-old patient with moderately impaired VA at baseline (0.7), but when adjusted for age-based norms, was considered to have normal VA.

The median starting dose of tovorafenib in the OPG subgroup was 420 mg/m^2^ (range 290-476 mg/m^2^). The median duration of tovorafenib treatment was 16 months, with 69% of patients (29/42) still on treatment at the data cutoff. Of the 35 patients in the analysis set (Supplementary Table S2), 24 (69%) were still on treatment at data cutoff; 3 (9%) had discontinued due to disease progression, 3 (9%) had discontinued due to AEs, 3 (9%) had withdrawn from the study, and 2 (6%) were on a drug holiday, of which 1 had restarted tovorafenib treatment due to disease progression.

### Imaging Outcomes

**Figure 1.**
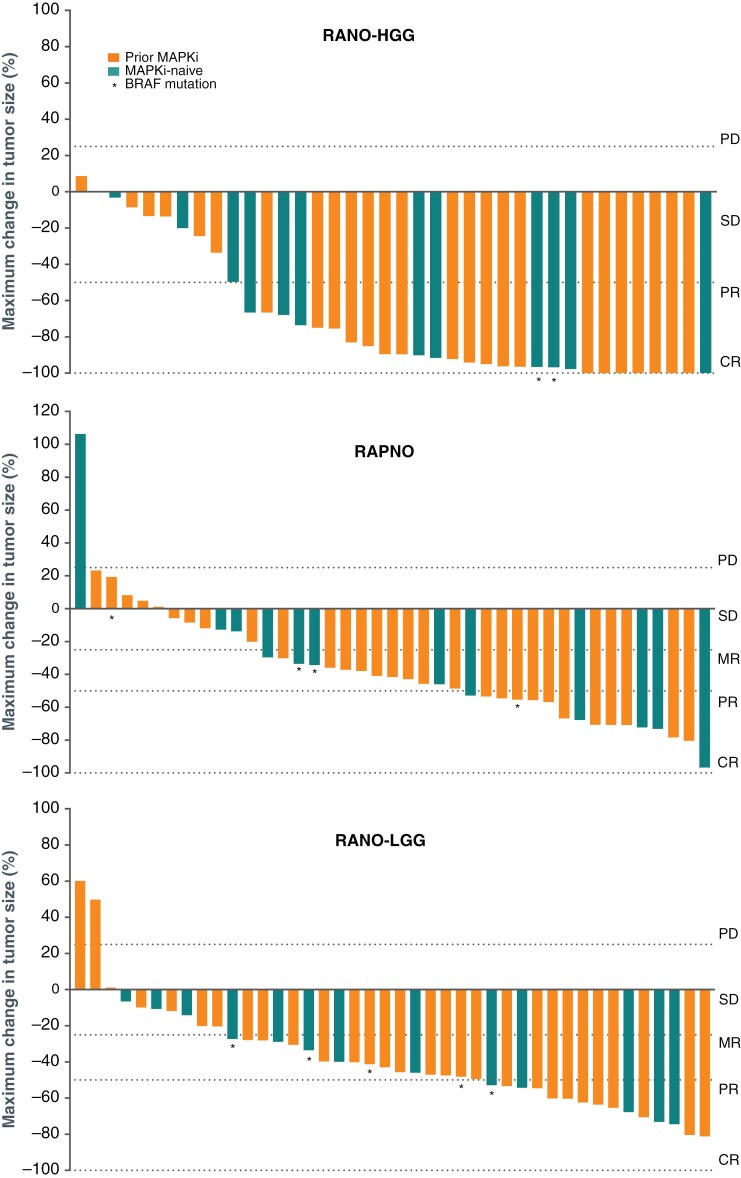
Best change in tumor size in patients with an OPG who were deemed evaluable for response by the independent radiology review committee according to RANO-HGG, RAPNO, and RANO-LGG criteria. Data for 1 patient was not included (RANO-HGG and RAPNO assessments) as they had no post-baseline contrast image. CR, complete response; HGG, high-grade glioma; LGG, low-grade glioma; MR, minor response; OPG, optic pathway glioma; PD, progressive disease; PR, partial response; RANO, Response Assessment in Neuro-Oncology; RAPNO, Response Assessment in Pediatric Neuro-Oncology; SD, stable disease.

The IRC deemed that 39 of the 42 patients in this analysis had measurable disease at baseline according to RANO-HGG criteria and were therefore evaluable for response. All 42 patients had measurable disease according to RAPNO and RANO-LGG criteria. The ORR and CBR (SD of any length of time) according to the contrast-enhancement-based RANO-HGG criteria were 64% and 95%, respectively, (Table 2). The ORRs (50% and 55%) and CBRs (88% and 90%) were similar according to the T2/FLAIR-based RAPNO and RANO-LGG criteria, respectively (both calculated by including MRs). Waterfall plots of best tumor response showed that most tumors had some degree of shrinkage as assessed both according to T1-weighted contrast-enhanced (RANO-HGG) and T2/FLAIR-based (RAPNO and RANO-LGG) criteria (Figure 1). Tumor shrinkage occurred both in OPGs harboring *BRAF* fusions and BRAF V600E mutations, and both in patients who had previously received mitogen-activated protein kinase inhibitor (MAPKi) therapy and patients who had not.

**Table 2.** Response by Radiological Assessment Criteria, Visual Acuity Response, and Clinical-Radiological Correlation

Response (IRC)	RANO-HGG *n* = 39	RAPNO *n* = 42	RANO-LGG *n* = 42
Overall response rate,^a^*n* (%)	25 (64)	21 (50)	23 (55)
95% CI	47-79	34-66	39-70
Clinical benefit rate,^a^*n* (%)			
SD of any length of time	37 (95)	37 (88)	38 (90)
SD ≥ 12 months	31 (79)	25 (60)	28 (67)
Best overall response, *n* (%)			
CR	7 (18)	0	0
PR	18 (46)	12 (29)	8 (19)
MR	n/a	9 (21)	15 (36)
SD	12 (31)	16 (38)	15 (36)
SD <12 months	6 (15)	12 (29)	10 (24)
SD ≥12 months	6 (15)	4 (10)	5 (12)
PD	2 (5)	5 (12)	3 (7)
NE	0	0	1 (2)
Median DOR, months (95% CI)^b^	16.8 (9.0-NR)	13.8 (11.3-NR)	14.4 (5.8-NR)
Median TTR, months (range)	5.5 (2.6-16.6)	5.5 (2.6-11.2)	5.5 (2.6-11.1)

Visual Acuity Response, *n* (%)^c^	Per Patient^d^ *n* = 35	Per Eye *n* = 52	
Preserved^e^	28 (80)	45 (87)	
Improved	11 (31)	14 (27)	
Stable	17 (49)	31 (60)	
Worsened	7 (20)	7 (13)	

Clinical-radiological correlation			
Per Patient^d^	RANO-HGG	RAPNO	RANO-LGG
	*n* = 35	*n* = 35	*n* = 34
Full concordance	13 (37)	16 (46)	13 (38)
Partial (positive) concordance *Improvement in 1 parameter; stability in the other*	16 (46)	11 (31)	17 (50)
Partial (negative) concordance *Worsening in 1 parameter; stability in the other*	2 (6)	7 (20)	3 (9)
Full discordance	4 (11)	1 (3)	1 (3)

Per Eye	*n* = 52	*n* = 52	*n* = 50
Full concordance	18 (35)	19 (37)	19 (38)
Partial (positive) concordance	26 (50)	20 (38)	25 (50)
Partial (negative) concordance	3 (6)	12 (23)	5 (10)
Full discordance	5 (10)	1 (2)	1 (2)

Abbreviations: BOR, best overall response; CBR, clinical benefit rate; CI, confidence interval; CR, complete response; DOR, duration of response; HGG, high-grade glioma; IRC, independent radiology review committee; LGG, low-grade glioma; MR, minor response; n/a, not applicable; NE, not evaluable; NR, not reached; ORR, overall response rate; PD, progressive disease; PR, partial response; RANO, Response Assessment in Neuro-Oncology; RAPNO, Response Assessment in Pediatric Neuro-Oncology; SD, stable disease; TTR, time to response; VA, visual acuity.

^a^ORR and CBRs for RAPNO-LGG and RANO-LGG included MRs (ie, ORR = CR + PR + MR; CBR = CR, PR, MR, or SD [calculated based on SD of any length of time and SD ≥ 12 months]).

^b^The exact 95% CIs were calculated using the Clopper-Pearson method.

^c^Seven patients are not included in the analysis; 4 had bilateral blindness and were not tested, 1 had no baseline assessment, 1 had no assessment after baseline, and 1 patient was deemed VA not evaluable at each assessment despite scores being entered.

^d^Includes 18 patients blind in 1 eye.

^e^Preserved includes patients with improved or stable visual acuity.

**Figure 2.**
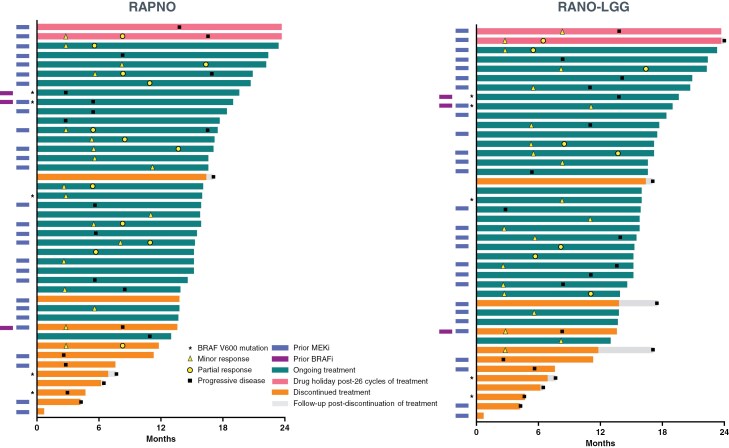
Swimlane plots of time to response and duration of therapy according to RAPNO and RANO-LGG criteria. In patients with confirmed responses, symbols indicate the start of response (MR or PR). If initial responses improved with continued treatment (from MR to confirmed PR), both the timepoint of the initial response and the timepoint that the response initially improved are marked accordingly. BRAFi, BRAF inhibitor; LGG, low-grade glioma; MEKi, MEK inhibitor; PR, partial response; MR, minor response; RANO, Response Assessment in Neuro-Oncology; RAPNO, Response Assessment in Pediatric Neuro-Oncology.

Duration of therapy and timing of response according to RAPNO and RANO-LGG criteria are shown in Figure 2. In 11 (26%) patients, an initial MR per RAPNO criteria was followed by a confirmed PR with continued treatment. Similarly, per RANO-LGG criteria, an initial MR in 6 (14%) patients was followed by a confirmed PR with continued treatment. Initial responses tended to be rapid, with a median TTR of 5.5 months according to each of the 3 assessment criteria (Table 2). Responses while patients were on treatment tended to be durable, with median DORs of 16.8 months (95% confidence interval [CI] 9.0-not reached [NR]), 13.8 months (95% CI 11.3-NR), and 14.4 months (95% CI 5.8-NR) per RANO-HGG, RAPNO, and RANO-LGG, respectively.

### VA Outcomes

**Figure 3.**
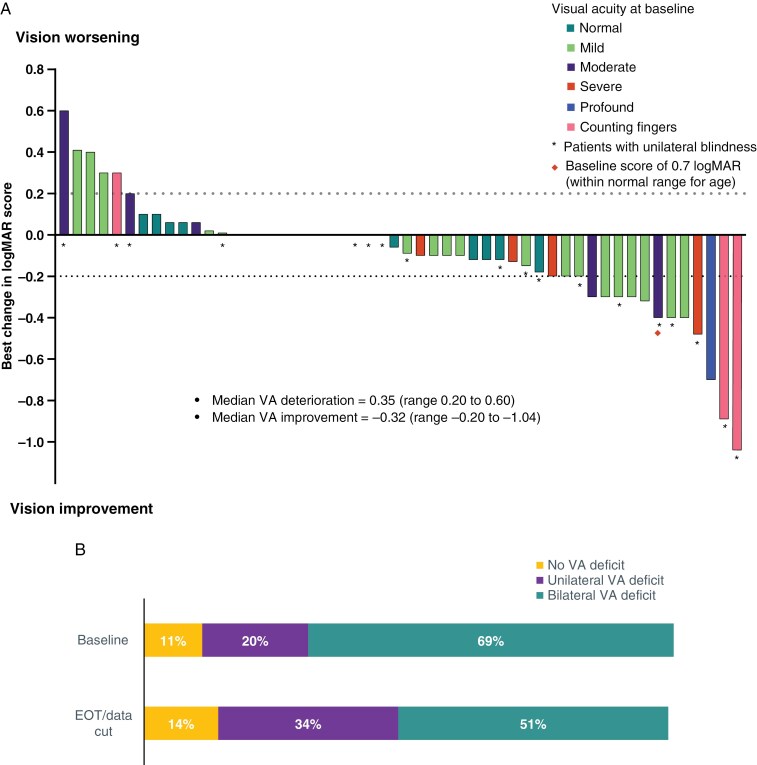
(A) Waterfall plot of best visual acuity change from baseline per eye (*n* = 52). Visual acuity progression was defined as an increase of ≥0.2 logMAR from baseline and visual acuity response as a decrease of ≥0.2 logMAR from baseline. (B) Visual morbidity per patient at the start and end of treatment/data cutoff (*n* = 35). EOT, end of treatment; logMAR, logarithm of the minimum angle of resolution; VA visual acuity.

Among the 35 patients with OPG with VA response data at baseline and at least 1 other timepoint, VA (per patient) improved in 11 (31%), remained stable (ie, no improvement or worsening) in 17 (49%), and worsened in 7 (20%) (Table 2). VA was therefore preserved (ie, improved or stable) in 80% of patients during treatment with tovorafenib. VA analysis per eye showed similar findings. Figure 3A shows a waterfall plot of the best change in VA per eye. The median VA deterioration was 0.35 (range 0.20-0.60) and the median VA improvement was −0.32 (range −0.20 to −1.04). The proportion of patients with preserved VA (per patient analysis) was similar in subgroups of patients with OPGs harboring *BRAF* fusions and BRAF V600E mutations, in those who had received prior MAPK inhibitor therapy and those who had not, and in patients who had received ≤3 prior lines of systemic therapy, and those who had received >3 (Supplementary Table S3). The per-eye analysis of VA response according to baseline VA showed that there were responders and preserved vision amongst all subgroups with impaired vision. VA improved in a proportion of eyes across most of these subgroups, and although numbers in each were small and not powered to make comparisons, no clear correlation to baseline VA was apparent (Supplementary Table S4). Stratifying the 52 eyes with VA response data (assessment at baseline and at least 1 other timepoint) into quartiles according to time from primary diagnosis suggested that vision was preserved in a high proportion of eyes across all quartiles (Supplementary Table S5). Notably, VA improvements were also seen (38%) in the quartile of eyes with the longest time from diagnosis.

### VA Response-Radiological Response Correlation

Concordance between VA (per patient and per eye) and radiological outcomes was broadly similar as assessed according to the different response criteria (Table 2). For RANO-HGG, full and partial concordance between these outcomes per patient was 37% and 46%, respectively, with only 4 cases (11%) fully discordant. Per eye was similar at 35% full concordance, 50% partial concordance, and only 5 cases (10%) fully discordant. For RAPNO, full and partial concordance per patient was 46% and 51%, respectively, with just 1 (3%) assessment fully discordant (per eye trends were similar at 37%, 62%, and 2% [1 case]), and for RANO-LGG, 38% and 59%, respectively, with 1 assessment (3%) fully discordant (per eye trends were similar at 38%, 60%, and 2% [1 case]).

Supplementary Table S6 shows the VA response per patient and per eye by best overall response, according to the different radiological assessment criteria. In patients with a radiological response to tovorafenib, vision per patient was improved in 32% of patients according to RANO-HGG, 47% of patients according to RAPNO, and 28% of patients according to RANO-LGG. Vision per patient was also improved in 30%, 23%, and 38% of patients with the best overall response of SD per RANO-HGG, RAPNO, and RANO-LGG, respectively. Figure 3B shows visual morbidity over time, which shows a decrease in the proportion of patients with a bilateral VA deficit from baseline to the end of treatment/data cutoff of 69%-51%.

### Safety

The most common treatment-emergent AEs (TEAEs; occurring in ≥20% of patients) and treatment-related AEs (TRAEs) occurring at grade ≥3 in at least 1 patient are listed in Table 3. Trends in TEAEs and TRAEs in the arm 1 OPG subgroup were similar to those reported for all patients in arms 1 and 2 (the safety population).^18^

**Table 3.** Treatment-Emergent and Treatment-Related Adverse Events (Arm 1 OPG Subgroup Safety Analysis Set, *n* = 42)

Preferred Term, *n* (%)	Treatment-Emergent AEs		Treatment-Related AEs	
	Any Grade	Grade ≥ 3	Any Grade	Grade ≥ 3
Patients with any AE	42 (100)	27 (64)	42 (100)	20 (48)
Hair color changes	36 (86)	0	36 (86)	0
Anemia	25 (60)	5 (12)	21 (50)	5 (12)
Fatigue	25 (60)	2 (5)	18 (43)	2 (5)
Elevated CPK	24 (57)	3 (7)	23 (55)	3 (7)
Vomiting	22 (52)	4 (10)	10 (24)	2 (5)
Headache	21 (50)	2 (5)	9 (21)	0
Maculopapular rash	21 (50)	4 (10)	20 (48)	4 (10)
Elevated LDH	17 (40)	0	12 (29)	0
Hypophosphatemia	17 (40)	0	14 (33)	0
Increased AST	16 (38)	2 (5)	14 (33)	2 (5)
Decreased appetite	16 (38)	1 (2)	10 (24)	1 (2)
Epistaxis	16 (38)	0	9 (21)	0
Pyrexia	16 (38)	1 (2)	4 (10)	0
Dry skin	15 (36)	0	14 (33)	0
Paronychia	15 (36)	1 (2)	14 (33)	1 (2)
COVID-19	14 (33)	0	0	0
Constipation	14 (33)	0	7 (17)	0
Nausea	14 (33)	0	5 (12)	0
Upper RTI	14 (33)	0	0	0
Weight decreased	12 (29)	2 (5)	9 (21)	0
Abdominal pain	11 (26)	0	6 (14)	0
Pain in extremity	11 (26)	0	4 (10)	0
Pruritus	11 (26)	0	10 (24)	0
Dermatitis acneiform	10 (24)	1 (2)	10 (24)	1 (2)
Eczema	10 (24)	1 (2)	9 (21)	1 (2)
Face edema	10 (24)	0	7 (17)	0
Increased ALT	9 (21)	0	7 (17)	0
Hypokalemia	9 (21)	3 (7)	6 (14)	2 (5)
Decreased growth velocity	7 (17)	2 (5)	7 (17)	2 (5)
Increased blood bilirubin	7 (17)	1 (2)	6 (14)	1 (2)
Viral eye infection	1 (2)	1 (2)	1 (2)	1 (2)
Hyponatremia	6 (14)	1 (2)	2 (5)	1 (2)
Lethargy	2 (5)	1 (2)	1 (2)	1 (2)
Erythematous rash	3 (7)	1 (2)	3 (7)	1 (2)
Follicular rash	1 (2)	1 (2)	1 (2)	1 (2)
Tumor hemorrhage	3 (7)	1 (2)	3 (7)	1 (2)

TEAEs, TRAEs, and laboratory abnormalities in ≥20% of patients and all TRAEs grade ≥3 occurring in ≥1 patient are reported. Patients are counted only once per event and are shown in the worst CTCAE grade that was reported for each event they experienced. Adverse events were coded according to MedDRA version 23.1 and graded according to CTCAE version 5.0.

Abbreviations: AEs, adverse events; ALT, alanine aminotransferase; AST, aspartate aminotransferase; COVID-19, coronavirus disease 2019; CPK, creatine phosphokinase; CTCAE, Common Terminology Criteria for Adverse Events; LDH, lactate dehydrogenase; MedDRA, Medical Dictionary for Regulatory Activities; RTI, respiratory tract infection; TEAE, treatment-emergent adverse event; TRAE, treatment-related adverse event.

Four patients (10%) had TEAEs leading to discontinuation, which included autoimmune hemolytic anemia, decreased growth velocity, tumor hemorrhage, and ventricular extrasystoles (1 patient each). The autoimmune hemolytic anemia was deemed unrelated to tovorafenib, whereas the other 3 events were considered to be TRAEs. More information on these events in the safety population (arms 1 and 2) has been published.^18^ Fifteen patients (36%) had TRAEs leading to dose reduction (median reduction, 1 level); the most common was decreased appetite (2 patients [5%]). Fifteen patients (36%) had TRAEs leading to dose interruption (median interruption, 7 days [1 week]); the most common were increased aspartate aminotransferase, maculopapular rash, and vomiting (2 patients [5%] each).

The incidence of ophthalmologic AESI in the FIREFLY-1 safety population and arm 1 OPG subgroup was also assessed, based on the MedDRA System Organ Class category of “Eye disorders,” excluding selected high-level group terms of “Congenital eye disorders (excl. glaucoma),” “Ocular neuromuscular disorders,” and “Ocular neoplasms.” Eight (6%) of 137 patients in the overall safety population had positively adjudicated ophthalmologic AESI (all grade 1-2), as detailed in Supplementary Table S7, with the most common being dyschromatopsia (deficiency in the perception of colors), experienced by 3 (2%) patients. There were no events of uveitis and no events involving the retina. AESI (all grade 1-2) deemed related to tovorafenib occurred in 3 (7%) of 42 patients in the OPG subgroup, including dyschromatopsia in 2 patients (5%) and glaucoma in 1 patient (2%) (Supplementary Table S7).

## Discussion

Sporadic OPGs appear to be more clinically challenging to treat than those associated with NF1. In particular, retrospective studies have suggested that sporadic OPGs are more likely to be symptomatic at diagnosis, patients with sporadic OPGs are more likely to receive treatment at diagnosis, and they are more likely to have severe long-term visual impairment than patients with NF1-associated OPGs.^27–30^

Given that curatively intentioned surgery for OPGs is only recommended for the rare patients with complete loss of vision and the tumor only located in front of the optic chiasm, sporadic OPGs must generally be managed as a chronic disease, often requiring multiple lines of systemic therapy over time to improve, preserve or at least minimize the loss of visual function. While radiotherapy may be effective for disease control in patients with OPG, the expected late toxicities mean use is avoided for children, adolescents, and young adults, although proton beam irradiation may limit the late toxicities.^10,31^ Consequently, chemotherapy, commonly comprising carboplatin (± vincristine) or vinblastine alone, is currently the preferred first-line treatment option, with the addition of bevacizumab to later lines of chemotherapy potentially improving visual outcomes.^12,13,32^ However, systematic reviews have suggested that the impact of systemic anticancer therapy in OPG on visual function is still unclear.^11,33^ Due to the frequent progressions during or after therapy, patients with sporadic OPG will often require multiple lines of therapy. New treatment is usually started if any deterioration in vision occurs, with the hope of preserving and stabilizing what remaining vision they have. However, patients can continue to have visual deterioration over time, with the concern that the longer the vision is affected, the less likely it is that there will be any recovery or improvement. Promisingly, preliminary data suggest that bevacizumab-based therapy may improve or stabilize vision in patients with OPG who have received multiple lines of prior treatment.^12,34,35^ Similarly, in the present analysis, we saw improved or stabilized VA during tovorafenib therapy in patients who had received multiple previous lines of therapy. Notably, improved VA was also seen in patients who had been diagnosed with tumors many years previously, in those who had received several prior lines of therapy, and in those who had previously been treated with drugs targeting MAPK pathway signaling. Interestingly, stratifying eyes according to time from primary diagnosis showed that VA improvements were still common in the quartile with the longest time from diagnosis. This suggests that tovorafenib treatment was able to achieve VA responses even in those with chronically impaired vision.

In the current subgroup analysis of the international, multicenter, single-arm phase 2 FIREFLY-1 trial, we demonstrated that tovorafenib monotherapy achieved clinically meaningful, rapid, and durable (while on treatment) tumor responses in children and young adults with *BRAF*-altered sporadic relapsed/refractory OPG. Similar to the recently published primary registrational analysis,^18^ tumor responses occurred in the OPG subgroup according to all 3 response assessment criteria, RANO-HGG (ORR, 64%), RAPNO (50%, including MRs), and RANO-LGG (55%, including MRs). Additionally, there was a consistent pattern of improved response over time according to both of the response criteria based on T2/FLAIR-weighted MRI sequences (RAPNO and RANO-LGG). The imaging responses to tovorafenib are particularly noteworthy in this OPG subgroup given that patients had received a median of 3 prior lines of systemic therapy, and more than two-thirds had previously received MEK and/or BRAF inhibitors.

Preservation of vision, often through stabilizing or reducing the size of tumors impacting optic nerve function, is an important treatment goal in this setting. Per patient, VA assessments showed that vision improved in 31% of patients, remained stable in 49%, and worsened in only 20%. Vision had therefore either improved or remained stable, that is, was preserved, in 80% of patients in the OPG subgroup during tovorafenib treatment. The improvement of VA (per patient) in 23%-38% of patients with a best overall response of SD (across assessment criteria) suggests that tumor shrinkage was not always needed in order to reach this important functional outcome, with vision perhaps improved in such patients through a reduction of pressure from the tumor on visual pathway components.

Preserved VA was seen regardless of *BRAF* alteration type (fusion vs mutation), whether or not patients had previously received MAPK inhibitor therapy, and whether they had received 3 or fewer or more than 3 prior lines of systemic therapy. VA generally remained stable or improved for the majority of eyes, regardless of the baseline VA (blind eyes excluded) (Supplementary Table S4). Clinically, it may be the case that even with stable tumor size, vision deteriorates with a longer time from initial diagnosis. In the present cohort treated with tovorafenib for OPGs, however, when stratifying eyes into quartiles according to time from primary diagnosis, vision was preserved during tovorafenib treatment in some patients who had been living with the disease for many years.

Published reports include visual outcomes in patients with sporadic and NF1-associated OPGs; in the case of the FIREFLY-1 trial, NF1 was an exclusion criterion. Of note, preclinical data suggest that tovorafenib monotherapy is unlikely to be effective in the treatment of *NF1* loss of function-associated OPGs.^36^ A recent systematic review on visual outcomes after treatment (radiotherapy, chemotherapy, or surgery) in pediatric patients with sporadic OPGs found 31 articles that met the search criteria; of those, 14 (45%) reported worsening outcomes after treatment, stable outcomes in 11 (35%), and improvement in 6 (19%).^37^ Results from reports on the individual treatment modalities (worsening, stable, and improvement, respectively) included: radiotherapy (8 studies): 3 (38%), 4 (50%), and 1 (13%); chemotherapy (14 studies): 5 (36%), 4 (29%), and 5 (36%); surgery (8 studies): 5 (63%), 3 (38%), and none. Visual decline at presentation, intraorbital optic nerve involvement, and intracranial hypertension requiring surgery were factors associated with poor outcomes. Some of the larger individual studies on the use of chemotherapy in that review, including the International Society of Paediatric Oncology Low-Grade Glioma (SIOP-LGG) 2004 trial UK cohort, which examined VA outcomes in a prospective trial of chemotherapy for OPG found that VA was stable (43%, 19/44) or improved (18%, 8/44) in 61% of the sporadic OPG cohort.^38^ Results were similar in the SIOP-LGG 2004 trial with 86% stable (61%, 59/96) or improved (25%, 24/96) (5.2 years median follow-up).^39^ The activity of tovorafenib in relation to improving vision in patients with heavily pretreated sporadic relapsed/refractory OPG compares favorably with results from studies of the effect of chemotherapy on VA. Further evidence that MAPK-pathway inhibition may be effective in this setting in relation to preserving vision derives from a phase 2 study evaluating the efficacy of the MEK1/2 inhibitor selumetinib in patients with recurrent/progressive optic pathway and hypothalamic LGG without NF1, which reported that VA was stable (68%, 13/19) or improved (21%, 4/19) in 89% of evaluable patients.^40^ In addition, in the randomized phase 2 trial of dabrafenib plus trametinib vs carboplatin plus vincristine, VA per eye in patients with BRAF V600-mutated suprasellar, chiasmatic, or hypothalamic tumors more frequently improved in those who received dabrafenib plus trametinib than in those who received chemotherapy (14 of 41 eyes examined [34%] vs 2 of 18 eyes examined [11%], respectively).^41^ Promising activity of bevacizumab in patients with OPG has also been reported. A study of 31 patients, 20 with sporadic OPG and 11 with NF1-associated OPG, who received bevacizumab monotherapy (35% previously treated with chemotherapy) reported that VA was stable in 56% (14/25) of evaluable patients and was improved in 32% (8/25), representing a visual preservation rate of 88%.^35^ A further study reported the outcome of bevacizumab-based treatment (third-line and beyond in 85% of patients) in 88 children with progressive pLGG (67 [76%] with sporadic tumors). In 65 evaluable patients with OPG, VA outcomes improved in 19 (29%) patients, remained stable in 32 (49%), and deteriorated in 14 (22%) patients, representing a visual preservation rate of 78%.^12^

In the current study, radiological and per-patient VA outcomes were fully concordant in 37%, 46%, and 38% of patients, and fully discordant in only 11%, 3%, and 3% of patients according to RANO-HGG, RAPNO, and RANO-LGG, respectively. The remaining assessments were partially concordant, with the largest group of patients having radiological responses and stable VA. Of note, for both RAPNO and RANO-LGG assessments, large improvements in VA occurred in some patients with only small maximal changes in tumor size.

The safety profile of tovorafenib monotherapy in the arm 1 OPG subgroup was similar to the previously reported safety profile in the safety analysis set of the combined population of treated patients in FIREFLY-1 arms 1 and 2.^18^ Whereas other drugs targeting the MAPK pathway can cause a variety of ophthalmological toxicities,^42,43^ no severe (grade ≥3) ophthalmological AEs related to tovorafenib therapy have been reported to date, including no uveitis and no events involving the retina. Similarly, ophthalmological toxicities were also not reported in the phase 2 trial of dabrafenib plus trametinib in patients with BRAF V600-mutated pLGG.^41^ Tumor hemorrhage was reported as a TRAE in 3 patients (7%) in the OPG subgroup. A retrospective study of 34 patients with OPG hemorrhages found that following treatment or observation, 20 experienced improvement in either visual function or neurological status, 2 experienced no improvement, and 6 patients died, with the clinical course unknown in 6. The study did not identify clear risk factors for hemorrhage in these patients.^44^

The main limitation of this analysis of VA outcomes in patients with OPG enrolled in arm 1 of the FIREFLY-1 trial is that it is a post hoc investigation, conducted in the absence of power calculations; analyses are consequently not powered to allow the drawing of definitive conclusions. In particular, the numbers of patients in subgroups defined by baseline characteristics are small, precluding the drawing of definitive conclusions about the effect of treatment in relation to VA in those subgroups. Also, a consistent VA testing method was not used for all participants, and in some instances, across assessments in individual participants. Transitioning between testing formats could therefore confound the interpretation of VA changes over time in this trial. We also note that the logMAR values allocated to very low vision, such as light perception, counting fingers, hand motion, etc., are estimates; therefore, the amount of logMAR change once vision was worse than 1.3 logMAR was also estimates. In addition, whether visual responses are durable off treatment has not yet been determined. Although visual field testing data were not regularly reported for participants in the current trial, we recognize that the addition of such data would have added to the robustness of our conclusions. The feasibility of such testing in pediatric patients with OPG has been demonstrated by Bennebroek et al,^34^ and the evaluation of visual fields in future studies in this setting would be appropriate. Despite the potential limitations, the current study nevertheless provides insight into the effectiveness of tovorafenib in this clinically important population of patients with sporadic relapsed/refractory OPG and in subgroups defined by various baseline characteristics.

In summary, in this group of children and adolescents with relapsed/refractory OPG, clinically meaningful, rapid, and durable (while on treatment) tumor responses (according to both contrast-enhancement-based and T2/FLAIR-weighted sequence-based response criteria) were achieved with tovorafenib monotherapy. No new safety signals were identified in this subgroup, with tovorafenib generally well tolerated and treatment discontinued due to TEAEs in only 10% of patients. There were no events of uveitis or events involving the retina, for example, retinal vein occlusion or central serous retinopathy. VA was preserved during treatment for 80% of patients and notably 31% demonstrated improved VA (per patient analysis). Tovorafenib may consequently offer an important new treatment option for patients with *BRAF*-altered, relapsed/refractory OPG.

## Supplementary material

Supplementary material is available online at *Neuro-Oncology* (https://academic.oup.com/neuro-oncology).

noae274_suppl_Supplementary_Tables

## Data Availability

The FIREFLY-1 trial protocol (confidential information redacted) has previously been published.^18^ The authors declare that all data supporting the findings of this subgroup analysis of the FIREFLY-1 trial are available within the article and Supplementary Information. Requests for full datasets will be considered after completion of the trial and analysis of the data, which is anticipated to be December 2026. To request individual participant data associated with any Day One Biopharmaceuticals clinical trial, please email clinical@dayonebio.com. All requests will be evaluated within 8 weeks.
