# Supplementary material for: Radiographic and visual response to the type II RAF inhibitor tovorafenib in children with relapsed/refractory optic pathway glioma in the FIREFLY-1 trial
Source: Neuro Oncol. 2024 Dec 19;27(5):1341–55. doi: 10.1093/neuonc/noae274 (PMC12187376; doi:10.1093/neuonc/noae274)
Supplement: noae274_suppl_Supplementary_Tables [file noae274_suppl_supplementary_tables.docx]

**Supplementary Material**

**Table S1.** Per patient VA testing methods used in FIREFLY-1 arm 1 OPG subgroup, n=35*

| **VA testing methods at any time point post-BL** |  | **VA testing method at BL** | | | |
| --- | --- | --- | --- | --- | --- |
|  |  | **TAC II** | **HOTV/ETDRS** | **Snellen** | **Other** |
|  | **TAC II** | 5 | 1 | 1 | - |
|  | **HOTV/ETDRS** | 2 | 4 | 1 | 1 (ATS HOTV) |
|  | **Snellen** | 1 | 3 | 7 | - |
|  | **Other** | - | - | 1  (VV logMAR) | 3 (2, Thomson crowded logMAR line;  1, Keeler Crowded logMAR) |
|  |  |  |  |  |  |
|  | **TAC II and Snellen** | - | 1 | - | - |
|  | **TAC II and HOTV/ETDRS** | - | - | 1 | - |
|  | **TAC II and other** | - | - | 1  (Near card) | - |
|  | **HOTV/ETDRS and Snellen** | - | - | - | 1 (Allen picture) |
|  | **TAC II, HOTV/ETDRS, and Snellen** | - | - | - | 1 (Near card with correction) |

*Includes 18 patients blind in one eye.

Grey shading indicates BL VA testing method did not change at any point post-BL.

- 19 (54%) patients were assessed with the same VA test method throughout the study
- 11 (31%) patients were assessed with one different VA test method any time point post-BL
- 4 (11%) patients were assessed with two different VA test methods any time point post-BL
- 1 (3%) patient was assessed with three different test methods at any time point post-BL

**Abbreviations:** ATS, Amblyopia Treatment Study; BL, baseline; ETDRS, Early Treatment Diabetic Retinopathy Study; logMAR, logarithm of the minimum angle of resolution; OPG, optic pathway glioma; TAC, Teller Acuity Cards^®^; VA, visual acuity; VV, VectorVision^®^.

**Table S2**. Patient characteristics and outcomes

| **Age at study entry** | **Sex** | ***BRAF* alteration status** | **Age at diagnosis** | **Prior lines systemic therapy, n** | **Duration of tovorafenib treatment, mo** | | **Treatment status** |  |  | **Outcomes following treatment** | | | | | |
| --- | --- | --- | --- | --- | --- | --- | --- | --- | --- | --- | --- | --- | --- | --- | --- |
|  |  |  |  |  |  |  |  | **Baseline VA (per eye)** | | **VA  (per eye)** | | **VA  (per patient)** | **Radiological** | | |
|  |  |  |  |  |  |  |  | **L** | **R** | **L** | **R** |  | **RANO-HGG** | **RAPNO** | **RANO-LGG** |
| 2 | M | Fusion | <1 | 4 | 15.2 | Ongoing | | 0.7* | Blind | Stable | Blind | Stable | CR | MR | MR |
| 4 | F | Fusion | 2 | 1 | 13.9 | Ongoing | | −0.1 | −0.1 | Stable | Stable | Stable | PR | MR | PR |
| 5 | M | Fusion | <1 | 2 | 15.2 | Ongoing | | 0.5 | 0.3 | Worsened | Stable | Worsened | PR | PR | PR |
| 5 | M | Fusion | 2 | 3 | 20.7 | Ongoing | | Blind | 0.18 | Blind | Stable | Stable | SD | MR | MR |
| 5 | M | Fusion | 2 | 2 | 17.2 | Ongoing | | Blind | 0.8 | Blind | Stable | Stable | PR | PR | PR |
| 6 | M | Fusion | 1 | 3 | 23.7 | d/c DH^†^ | | 0.2 | Blind | Improved | Blind | Improved | PR | PR | PR |
| 6 | M | Fusion | <1 | 7 | 18.4 | Ongoing | | Blind | 1.84 | Blind | Improved | Improved | SD | SD | SD |
| 6 | M | Fusion | 1 | 3 | 16.4 | d/c AE | | Blind | 2 | Blind | Improved | Improved | SD | SD | SD |
| 7 | M | Mutation | 5 | 1 | 4.7 | d/c PD | | 1.3 | 0.4 | Stable | Worsened | Worsened | PD | PD | PD |
| 7 | F | Fusion | 4 | 2 | 13.8 | d/c AE | | 1.4 | 0 | Improved | Worsened | Worsened | PR | SD | SD |
| 7 | F | Mutation | 5 | 1 | 6.9 | d/c parent W/D | | 0.2 | 0.49 | Stable | Worsened | Worsened | SD | SD | SD |
| 7 | F | Fusion | 5 | 2 | 16 | Ongoing | | 0.7 | 0.3 | Improved | Stable | Improved | PR | PR | SD |
| 7 | F | Fusion | 3 | 2 | 23.3 | Ongoing | | Blind | 0 | Blind | Stable | Stable | CR | PR | PR |
| 8 | M | Mutation | 1 | 3 | 19.6 | Ongoing | | 1.2 | 1.1 | Stable | Stable | Stable | SD | PD | SD |
| 8 | F | Fusion | 2 | 5 | 17.5 | Ongoing | | 0.4 | 0.48 | Improved | Improved | Improved | PD | PR | NE |
| 8 | M | Fusion | <1 | 5 | 22.4 | Ongoing | | 0.49 | Blind | Stable | Blind | Stable | CR | SD | SD |
| 8 | M | Fusion | 1 | 5 | 15.5 | Ongoing | | 0.8 | Blind | Stable | Blind | Stable | CR | SD | MR |
| 8 | F | Mutation | 4 | 2 | 16 | Ongoing | | 0.2 | Blind | Stable | Blind | Stable | SD | MR | MR |
| 9 | F | Fusion | 1 | 3 | 11.8 | d/c AE | | 0.4 | 0.3 | Improved | Stable | Improved | PR | PR | MR |
| 9 | M | Fusion | <1 | 9 | 20.9 | Ongoing | | 0.3 | Blind | Improved | Blind | Improved | PR | PR | SD |
| 9 | M | Fusion | 3 | 9 | 13.7 | Ongoing | | 0 | Blind | Stable | Blind | Stable | SD | SD | SD |
| 10 | M | Mutation | 8 | 2 | 19 | Ongoing | | 0.3 | 1 | Improved | Improved | Improved | SD | SD | MR |
| 10 | M | Fusion | 3 | 1 | 17.7 | Ongoing | | 0 | 0 | Stable | Stable | Stable | PR | PD | MR |
| 10 | F | Fusion | <1 | 8 | 16.6 | Ongoing | | 1.1 | Blind | Improved | Blind | Improved | SD | MR | SD |
| 10 | M | Fusion | <1 | 6 | 15.9 | Ongoing | | 0.8 | Blind | Worsened | Blind | Worsened | CR | SD | PD |
| 11 | F | Fusion | <1 | 5 | 11.3 | d/c pt W/D | | 0.56 | 0.32 | Worsened | Stable | Worsened | SD | PD | PD |
| 12 | F | Fusion | 5 | 4 | 17.2 | Ongoing | | 0.06 | 1 | Stable | Stable | Stable | CR | PR | PR |
| 12 | M | Fusion | 3 | 6 | 15.3 | Ongoing | | Blind | 0.5 | Blind | Improved | Improved | PR | PR | PR |
| 13 | F | Fusion | 12 | 1 | 6.2 | d/c PD | | 0.06 | 0.06 | Stable | Stable | Stable | SD | SD | SD |
| 13 | F | Fusion | 7 | 3 | 7.6 | d/c PD | | 0.3 | −0.1 | Stable | Stable | Stable | PR | PD | SD |
| 13 | F | Fusion | 4 | 4 | 13.8 | Ongoing | | 0.3 | 2.3 | Improved | Stable | Improved | PR | MR | MR |
| 14 | M | Fusion | 1 | 4 | 23.7 | d/c DH | | 0.49 | Blind | Stable | Blind | Stable | PR | SD | MR |
| 14 | M | Fusion | <1 | 5 | 4.2 | d/c parent W/D | | Blind | 0.7 | Blind | Worsened | Worsened | PD | SD | SD |
| 15 | M | Fusion | 9 | 3 | 13 | Ongoing | | 1.7 | Blind | Stable | Blind | Stable | PR | SD | MR |
| 16 | F | Fusion | 14 | 2 | 22.3 | Ongoing | | 0 | 0 | Stable | Stable | Stable | PR | PR | PR |

Month for age was not collected in the EDC, so age at diagnosis is a rough estimate and may not be entirely accurate (i.e., may be off by 1 year). Was calculated backward from age at C1D1 (baseline) and the patient’s diagnosis date.
*Left eye was considered “normal” at baseline when adjusted for age. ^†^Progressed while on a DH; currently being retreated.
**Abbreviations:** AE, adverse event; CR, complete response; d/c, discontinued; DH, drug holiday; EDC, Electronic Data Capture; F, female; HGG, high grade glioma; LGG, low-grade glioma; M, male; mo, months; MR. minor response; PD, progressive disease; pt, patient; PR, partial response; RANO, Response Assessment in Neuro-Oncology; RAPNO; Response Assessment in Pediatric Neuro-Oncology; SD, stable disease; VA, visual acuity; W/D, withdraw.

**Table S3**. Visual acuity response (per patient and per eye) during treatment according to baseline characteristics^a^

|  |  | ***BRAF* alteration status** | | **Prior MAPKi targeted therapy** | | **Number of prior lines of  systemic therapy** | |
| --- | --- | --- | --- | --- | --- | --- | --- |
|  |  | **Fusion** | **V600E mutation** | **MAPKi-naive** | **Prior MAPKi** | **≤3** | **>3** |
| **Visual acuity response per patient (n=35^b^)** | | | | | | | |
| Preserved^c^ |  | 25 (83) | 3 (60) | 9 (75) | 19 (83) | 16 (80) | 12 (80) |
| Improved |  | 10 (33) | 1 (20) | 3 (25) | 8 (35) | 5 (25) | 6 (40) |
| Stable |  | 15 (50) | 2 (40) | 6 (50) | 11 (48) | 11 (55) | 6 (40) |
| Worsened |  | 5 (17) | 2 (40) | 3 (25) | 4 (17) | 4 (20) | 3 (20) |
| **Visual acuity response per eye (n=52)** | | | | | | | |
| Preserved^c^ |  | 38 (88) | 7 (78) | 17 (85) | 28 (87) | 29 (88) | 16 (84) |
| Improved |  | 12 (28) | 2 (22) | 3 (15) | 11 (34) | 7 (21) | 7 (37) |
| Stable |  | 26 (60) | 5 (56) | 14 (70) | 17 (53) | 22 (67) | 9 (47) |
| Worsened |  | 5 (12) | 2 (22) | 3 (15) | 4 (13) | 4 (12) | 3 (16) |

Data are n (%).

^a^Seven patients are not included in the analysis; four had bilateral blindness and were not tested, one had no baseline assessment, one had no assessment after baseline, and one patient was deemed VA not evaluable at each assessment despite scores being entered.

^b^Includes 18 patients who were blind in one eye.

^c^Preserved includes patients with improved or stable visual acuity.

**Abbreviation:** MAPKi, mitogen-activated protein kinase inhibitor.

**Table S4**. Visual acuity response (per eye) by baseline visual acuity status^a^

|  | | **Degree of baseline visual impairment** (logMAR range) | | | | |  |  |  |  |
| --- | --- | --- | --- | --- | --- | --- | --- | --- | --- | --- |
|  |  | Normal vision  (up to 0.19) | Mild  (0.2 to 0.5) | Moderate  (0.6 to 0.9) | Severe  (1.0 to 1.3) | Profound  (1.4 to 1.6) | Counting  fingers  (1.7 to 2.0) | Hand motion (2.1 to 2.4) | Light perception (2.5 to 2.9) | No light perception  (≥3.0) |
| **Visual acuity response per eye (n=52)** | | | | | | |  |  |  |  |
| Preserved^b^ | | 14 (93) | 17 (85) | 3 (50) | 6 (100) | 1 (100) | 3 (100) | 1 (100) | 0 | 0 |
| Improved | | 0 | 8 (40) | 1 (17) | 2 (33) | 1 (100) | 2 (67) | 0 | 0 | 0 |
| Stable | | 14 (93) | 9 (45) | 2 (33) | 4 (67) | 0 | 1 (33) | 1 (100) | 0 | 0 |
| Worsened | 1 (7) | 3 (15) | 3 (50) | 0 | 0 | 0 | 0 | 0 | 0 |  |

Data are *n* (%).

^a^Seven patients are not included in the analysis; four had bilateral blindness and were not tested, one had no baseline assessment and one had no assessment after baseline, and one patient was deemed VA not evaluable at each assessment despite scores being entered.

^b^Preserved includes patients with improved or stable visual acuity.

**Abbreviation:** logMAR, logarithm of the minimum angle of resolution.

**Table S5.** Visual acuity response (per patient and per eye) by time from primary diagnosis quartiles^a^

|  | **Quartile: time from primary diagnosis, years** | | | |
| --- | --- | --- | --- | --- |
|  | **Q1: 0.9–2.6** | **Q2: 2.6–5.8** | **Q3: 5.8–7.8** | **Q4: 7.8–13.8** |
| **Visual acuity response per patient (n=35^b^)** | | | | |
| Preserved^c^ | 6 (86) | 7 (70) | 8 (100) | 7 (70) |
| Improved | 2 (29) | 2 (20) | 2 (25) | 5 (50) |
| Stable | 4 (57) | 5 (50) | 6 (75) | 2 (20) |
| Worsened | 1 (14) | 3 (30) | 0 | 3 (30) |
| **Visual acuity response per eye (n=52)** | | | | |
| Preserved^c^ | 12 (92) | 10 (77) | 13 (100) | 10 (77) |
| Improved | 3 (23) | 3 (23) | 3 (23) | 5 (38) |
| Stable | 9 (69) | 7 (54) | 10 (77) | 5 (38) |
| Worsened | 1 (8) | 3 (23) | 0 | 3 (23) |

Data are n (%). Quartiles for time from primary diagnosis were defined from the per eye analysis.

^a^Seven patients are not included in the analysis; four had bilateral blindness and were not tested, one had no baseline assessment and one had no assessment after baseline, and one patient was deemed VA not evaluable at each assessment despite scores being entered.

^b^Includes 18 patients who were blind in one eye.

^c^Preserved includes patients with improved or stable visual acuity.

**Abbreviation:** Q, quartile.

**Table S6.** Visual acuity response (per patient and per eye) by best overall response according to different radiological assessment criteria^a^

|  | **Best overall response** | | | | | | | |  |
| --- | --- | --- | --- | --- | --- | --- | --- | --- | --- |
|  | **CR/PR/MR^b^** | **CR** | **PR** | **MR^c^** | **SD** | **PD** | **NE** | **Total** |  |
| **Visual acuity response per patient (n=35^c^)** | | | | | | | | |  |
|  | **RANO-HGG** | | | | | | | |  |
| Preserved^d^ | 18 (86) | 5 (83) | 13 (87) | - | 9 (82) | 1 (33) | 0 | 28 (80) |  |
| Improved | 6 (29) | 0 | 6 (40) | - | 4 (36) | 1 (33) | 0 | 11 (31) |  |
| Stable | 12 (57) | 5 (83) | 7 (47) | - | 5 (45) | 0 | 0 | 17 (49) |  |
| Worsened | 3 (14) | 1 (17) | 2 (13) | - | 2 (18) | 2 (67) | 0 | 7 (20) |  |
| Total | 21 | 6 | 15 | - | 11 | 3 | 0 | 35 (100) |  |
|  | **RAPNO** | | | | | | | |  |
| Preserved^d^ | 16 (94) | 0 | 10 (91) | 6 (100) | 9 (69) | 3 (60) | 0 | 28 (80) |  |
| Improved | 8 (47) | 0 | 6 (55) | 2 (33) | 3 (23) | 0 | 0 | 11 (31) |  |
| Stable | 8 (47) | 0 | 4 (36) | 4 (67) | 6 (46) | 3 (60) | 0 | 17 (49) |  |
| Worsened | 1 (6) | 0 | 1 (9) | 0 | 4 (31) | 2 (40) | 0 | 7 (20) |  |
| Total, n | 17 | 0 | 11 | 6 | 13 | 5 | 0 | 35 (100) |  |
|  | **RANO-LGG** | | | | | | | |  |
| Preserved^d^ | 17 (94) | 0 | 7 (88) | 10 (100) | 10 (77) | 0 | 1 (100) | 28 (80) |  |
| Improved | 5 (28) | 0 | 2 (25) | 3 (30) | 5 (38) | 0 | 1 (100) | 11 (31) |  |
| Stable | 12 (67) | 0 | 5 (63) | 7 (70) | 5 (38) | 0 | 0 | 17 (49) |  |
| Worsened | 1 (6) | 0 | 1 (13) | 0 | 3 (23) | 3 (100) | 0 | 7 (20) |  |
| Total, n | 18 | 0 | 8 | 10 | 13 | 3 | 1 | 35 (100) |  |
| **Visual acuity response per eye (n=52)** | | | | | | | | |  |
|  | | **RANO-HGG** | | | | | | | |
| Preserved^d^ | | 28 (90) | 6 (86) | 22 (92) |  | 14 (88) | 3 (60) | 0 | 45 (87) |
| Improved | | 7 (23) | 0 | 7 (29) | - | 5 (31) | 2 (40) | 0 | 14 (27) |
| Stable | | 21 (68) | 6 (86) | 15 (63) | - | 9 (56) | 1 (20) | 0 | 31 (60) |
| Worsened | | 3 (10) | 1 (14) | 2 (8) | - | 2 (13) | 2 (40) | 0 | 7 (13) |
| Total | | 31 | 7 | 24 | - | 16 | 5 | 0 | 52 (100) |
|  | | **RAPNO** | | | | | | | |
| Preserved^d^ | | 24 (96) | 0 | 16 (94) | 8 (100) | 13 (76) | 8 (80) | 0 | 45 (87) |
| Improved | | 9 (36) | 0 | 7 (41) | 2 (25) | 5 (29) | 0 | 0 | 14 (27) |
| Stable | | 15 (60) | 0 | 9 (53) | 6 (75) | 8 (47) | 8 (80) | 0 | 31 (60) |
| Worsened | | 1 (4) | 0 | 1 (6) | 0 | 4 (24) | 2 (20) | 0 | 7 (13) |
| Total, n | | 25 | 0 | 17 | 8 | 17 | 10 | 0 | 52 (100) |
|  | | **RANO-LGG** | | | | | | | |
| Preserved^d^ | | 25 (96) | 0 | 11 (92) | 14 (100) | 16 (84) | 2 (40) | 2 (100) | 45 (87) |
| Improved | | 6 (23) | 0 | 2 (17) | 4 (29) | 6 (32) | 0 | 2 (100) | 14 (27) |
| Stable | | 19 (73) | 0 | 9 (75) | 10 (71) | 10 (53) | 2 (40) | 0 | 31 (60) |
| Worsened | | 1 (4) | 0 | 1 (8) | 0 | 3 (16) | 3 (60) | 0 | 7 (13) |
| Total, n | | 26 | 0 | 12 | 14 | 19 | 5 | 2 | 52 (100) |

Data are n (%) unless otherwise stated.

^a^Seven patients are not included in the analysis; four had bilateral blindness and were not tested, one had no baseline assessment and one had no assessment after baseline, and one patient was deemed VA not evaluable at each assessment despite scores being entered.

^b^MR classification only applies to RAPNO and RANO-LGG criteria.

^c^Includes 18 patients who were blind in one eye.

^d^Preserved includes patients with improved or stable visual acuity.

**Abbreviations:** CR, complete response; HGG, high-grade glioma; LGG, low-grade glioma; MR, minor response; NE, not evaluable; PD, progressive disease; PR, partial response; RANO, Response Assessment in Neuro-Oncology; RAPNO, Response Assessment in Pediatric Neuro-Oncology; SD, stable disease.

**Table S7.** Positively adjudicated ophthalmologic AESI^a^ in the safety population

| Category  **Preferred Term, n (%)** | Safety analysis set (arm 1 + arm 2, *n*=137) | | Arm 1 OPG subgroup *n*=42 | |
| --- | --- | --- | --- | --- |
|  | Any grade | Grade ≥3 | Any grade | Grade ≥3 |
| Ophthalmologic events | 8 (6) | 0 | 3 (7) | 0 |
| Vision blurred | 1 (1) | 0 | 0 | 0 |
| Dyschromatopsia | 3 (2) | 0 | 2 (5) | 0 |
| Corneal edema | 1 (1) | 0 | 0 | 0 |
| Glaucoma | 1 (1) | 0 | 1 (2) | 0 |
| Photopsia | 1 (1) | 0 | 0 | 0 |
| Episcleritis | 1 (1) | 0 | 0 | 0 |

^a^Categorized according to the Medical Dictionary for Regulatory Activities Version 23.1 and graded according to the Common Terminology Criteria for Adverse Events Version 5.0.

Note: Only events positively adjudicated as ophthalmologic events are presented.

**Abbreviation:** AESI, adverse events of special interest.
